# Benefits, side effects, and uses of *Hericium erinaceus* as a supplement: a systematic review

**DOI:** 10.3389/fnut.2025.1641246

**Published:** 2025-09-01

**Authors:** Arya Menon, Ayesha Jalal, Zara Arshad, Faisal A. Nawaz, Rahul Kashyap

**Affiliations:** ^1^College of Medicine, Mohammed Bin Rashid University of Medicine and Health Sciences, Dubai, United Arab Emirates; ^2^Global Remote Research Scholars Program, Princeton Junction, NJ, United States; ^3^Al Amal Psychiatric Hospital, Emirates Health Services, Dubai, United Arab Emirates; ^4^Department of Research, WellSpan Health, York, PA, United States

**Keywords:** *Hericium erinaceus*, Lion’s mane mushroom, neuroprotection, neurodegenerative diseases, anti-tumor, dietary supplement, cognitive function, mood disorder

## Abstract

**Introduction:**

*Hericium erinaceus* (HE), commonly known as the Lion’s Mane mushroom, is an edible, medicinal fungus containing bioactive polysaccharides. It shows promising properties, including neuroprotective, anti-tumor, antioxidant, anti-proliferative, and anti-inflammatory effects. The mushroom’s bioactive chemical components, derived from its fruiting bodies and mycelia (erinacines), demonstrate promising neural-stimulating activity. This systematic review investigates existing literature on the clinical use of HE, outlining its benefits and side effects related to neural stimulation, apoptotic activity, the abundance of short-chain fatty acids (SCFAs)-producing microbiota, and its role in mood dysregulation, with the aim of establishing a safety profile for the supplement.

**Materials and methods:**

A systematic literature search was conducted following the Preferred Reporting Items for Systematic Reviews and Meta-Analyses (PRISMA) guidelines. PubMed was searched for relevant, peer-reviewed articles published between January 2000 and June 2024. To address the risk of quality bias, the ROBIS tool was used to eliminate bias and ensure the quality of the included studies. This systematic review is registered on PROSPERO (ID: CRD42024571250).

**Result:**

This review includes results from five randomized controlled trials (RCTs), 15 laboratory studies, three pilot clinical trials (PCTs), one cohort study, one case report, and one computer analysis. The RCTs and PCTs assessed cognitive improvements in participants with and without dementia. Mini-Mental State Examination scores from one RCT and one PCT showed a combined weighted mean increase of 1.17 in the intervention group. *In vitro* laboratory studies on cancer and cell apoptosis, focusing on leukemia and gastric cancer cells, found that isolated erinacine A from the mycelium of HE inhibited the invasiveness of MKN28 and TSGH 9201 cells and activated caspase pathways. HE also increased gut microbiota diversity and the abundance of SCFA-producing bacteria, thereby reducing inflammation and protecting gut health. Additionally, HE enhanced pro-BDNF and BDNF production, promoted hippocampal neurogenesis, improved behavior, and reduced symptoms of depression, anxiety, binge eating, and sleep disorders.

**Discussion:**

HE is effective in neuroprotection, enhancing cognitive function, preventing and alleviating cancer, promoting gut health, and improving symptoms of anxiety and depression. Although commonly unreported, potential side effects of HE include stomach discomfort, headache, and allergic reactions.

**Systematic review registration:**

https://www.crd.york.ac.uk/PROSPERO/view/CRD42024571250.

## 1 Introduction

*Hericium erinaceus* (HE) is a popular and traditional edible mushroom native to North America, Europe, and Asia. Deeply rooted in traditional Chinese medicine, HE is widely cultivated and valued as a medicinal mushroom, primarily for its neuroprotective and immune-boosting properties (1). Erinacines derived from HE have been shown to stimulate the synthesis of neurotrophins such as nerve growth factor (NGF) and brain-derived neurotrophic factor (BDNF), aiding in neuronal activity and survival (2).

Lion’s mane mushroom contains multiple phytochemicals, such as *β*-glucan and hericenones, which promote NGF synthesis *in vitro.* Erinacines have also been found to prevent neuronal death, promote neurite outgrowth, and support the maintenance of neuronal functions (3). Additionally, these mushrooms are rich in vitamins B1, B2, and B3, as well as essential minerals, such as manganese, zinc, and potassium. Erinacines and hericenones derived from the fruiting body of HE have been shown to affect the autonomic nervous system through the stimulation of NGF in astrocytes, cells essential for neural survival (4). This mechanism supports the potential clinical benefits of HE in mood-regulating disorders such as anxiety and depression (5).

Neurodegenerative disorders such as dementia are reported to be the seventh leading cause of death, dependency, and disability among the global senior population. Alarmingly, more than 55 million people worldwide have dementia, 60% of whom reside in low- and medium-income countries, as reported by the World Health Organization (WHO) (6).

Studies have also indicated that HE mushroom extracts can inhibit the proliferation of various cancer cells, including liver cancer (HepG2 and Huh-7), colon cancer (HT-29), gastric cancer (NCI-87 cells) (7), breast cancer (MCF-7), cervical cancer (HeLa) (8), human acute promyelocytic leukemia (HL-60), and lung fibroblast cells (HEL-299) (9).

In this systematic review, we identified, evaluated, and summarized the published literature on the benefits and side effects of HE. This article aims to provide a comprehensive overview of the benefits of taking HE as a regular supplement. Additionally, we examined the potential adverse effects to establish a safety profile for HE.

## 2 Materials and methods

**Figure 1:**
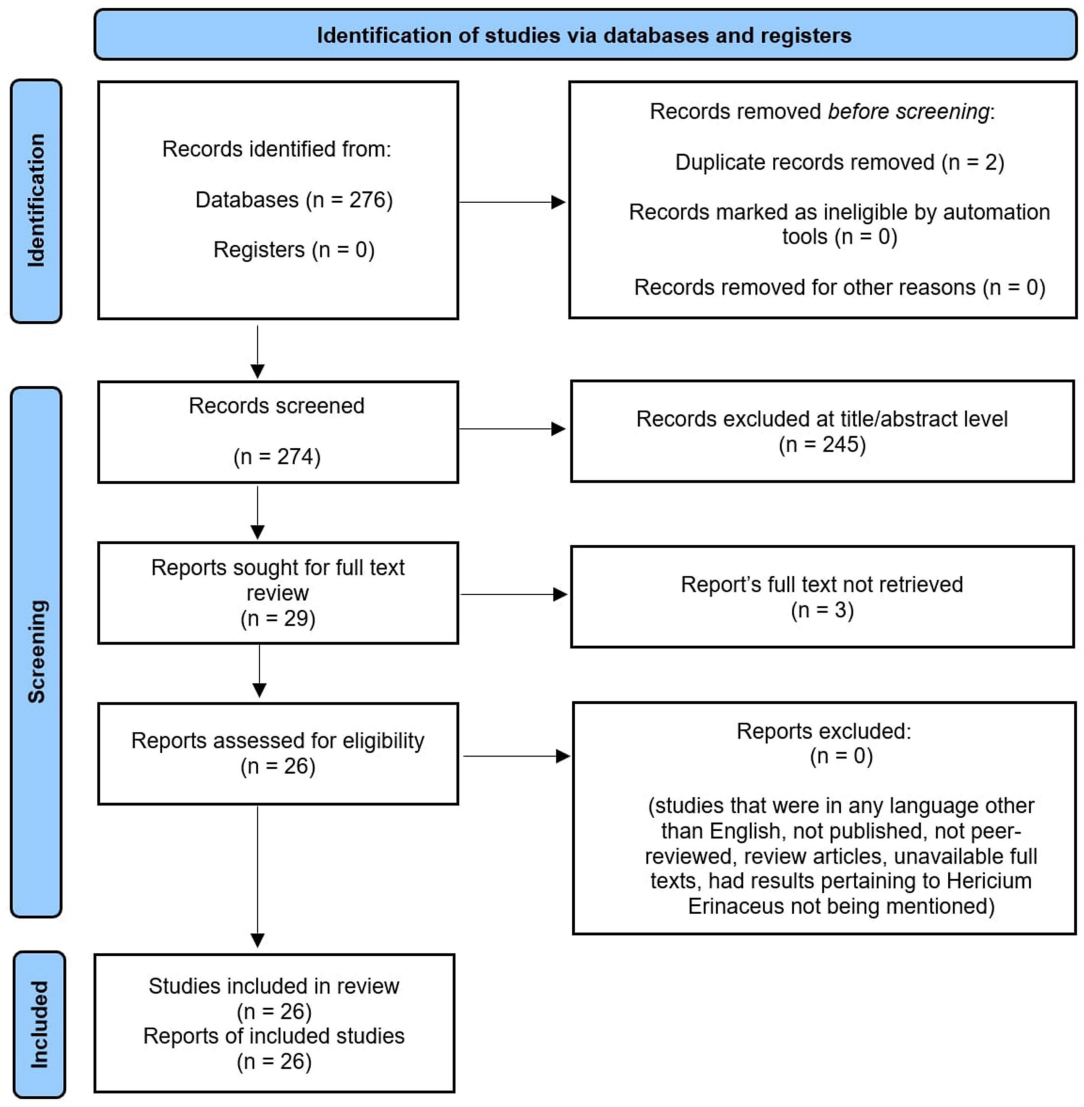
Depicts the PRISMA flowchart for identifying relevant studies.

A total of 26 studies published between 2000 and 2024 were included in this systematic review. The search strategy primarily used the keywords “*Hericium erinaceus*” and “Lion’s Mane.” A comprehensive search of the PubMed database was conducted to identify full-length articles that met the inclusion criteria, followed by a manual review of the reference lists of the selected articles. The review process followed the PRISMA guidelines to ensure transparent and comprehensive reporting, as demonstrated by Figure 1.

### 2.1 Search strategy

We used Boolean terminology to search PubMed for relevant articles published between 2000 and 2024. Some keywords we included in our search strategy string included “*Hericium erinaceus,*” “Lion’s Mane,” “Yambushitake,” “benefit,” “health,” “mental health,” “neur*,” “stomach,” “supplement,” and “anticancer.”

### 2.2 Eligibility criteria

Articles were included if they met the inclusion criteria: (1) peer-reviewed, (2) published in English, (3) full-text available, and (4) published between 2000 and 2024. Articles were excluded based on the following exclusion criteria: (1) written in a language other than English, (2) not published, (3) not peer-reviewed, (4) review articles, other systematic reviews, or book chapters, (5) unavailable full texts, and (6) no mention of benefits or side effects related to *Hericium erinaceus*.

### 2.3 Data extraction

Data extraction and collection were performed in accordance with the Preferred Reporting Items for Systematic Review and Meta-Analyses (PRISMA) guidelines (10). All articles retrieved from the PubMed search were initially exported to Rayaan (11), and duplicates were removed. Two authors independently and blindly screened the titles and abstracts to identify studies meeting the inclusion criteria, resolving any conflicts through discussion. Relevant data from the included articles were then imported into Google Sheets.

### 2.4 Data analysis

We analyzed pooled data from the intervention and placebo groups, reporting the number and percentages of participants in each group. Pooled odds ratios with 95% CI (*p*-values) were calculated for both efficacy/benefits and side effects. Additionally, we reported descriptive demographics and side effects from the included case reports. Microsoft Excel was used to extract data from the selected studies and to assess the risk of bias.

### 2.5 Risk of bias assessment

The Cochrane RoB-2, ROBINS-I, Newcastle-Ottawa Scale (NOS), IHE Case Report Quality Appraisal Tool, and SYRCLE Risk of Bias tool identified concerns regarding the study’s eligibility criteria (12–16). These tools were used to assess relevance, evaluate the review process, and determine the risk of bias. Two independent researchers assessed the selected studies and assigned a quality rating.

## 3 Results

### 3.1 Study selection

The systematic search yielded a total of 276 studies from the PubMed database. Following the exclusion of 2 duplicates, 274 articles remained for screening. These were further filtered through titles and abstracts. The screening of titles and abstracts yielded 130 articles that met the inclusion criteria, consisting of human- and animal-based studies. At this stage, animal studies were excluded, leaving 54 human studies. After further evaluation of the full articles and study designs, 26 full-text articles met the inclusion criteria for this systematic review. Furthermore, a descriptive summary of all human studies (Table 1) and laboratory and computer-based studies (Table 2) was created to explore the pharmacological and biological activities of HE.

**Table 1:** Patient and study characteristics (including all human studies).

Study	Design	Duration	Sample	Age (years)	Gender	Status	Study Focus	Treatment	Side effects	Scales	References
Saitsu et al. (2019), Japan	RCT	84 days	N = 31 I = 16 C = 15	I = 61.8 ± 1.7 C = 60.8 ± 2.2	M = 11 F = 20	Healthy	Improving cognitive function	HE supplements contained 0.8 g of fruiting powder from HE	None reported	MMSE, Benton visual retention test, S-PA	(19)
Nagano et al. (2010), Japan	RCT	28 days	N = 26 I = 12 C = 14	I = 41.3 ± 5.6 C = 38.4 ± 4.9	M = 0 F = 26	Healthy	Reduction of depression and anxiety	4 HE cookies daily containing 0.5 g of the powdered fruiting body of HE each	Epi-menorrhea	KMI, CES-D. PSQI. ICI	(5)
Mori et al. (2009), Japan	RCT	154 days	N = 30 I = 15 C = 15	N = 50 to 80	M = 15 F = 15	Patient	Improving mild cognitive impairment	4 tablets containing 250 mg three times a day	Stomach discomfort and diarrhea	HDS-R	(17)
La Monica et al. (2023), USA	RCT	7–14 days	N = 40 I = 40 C = 40	N = 34.0 ± 9.5	M = 18 F = 22	Healthy	Improving cognitive performance	A single dose of 1 g of Nordic-grown Lion’s Mane	Headache	Go/No-go, Serial Sevens, N-Back, VAS, SHS	(18)
Vigna et al. (2019), Italy	RCT	120 days	N = 77 I = 40 C = 37	N = 53.2 ± 0.7	M = 15 F = 62	Patient	Improving mood and sleep disorders	3 HE capsules a day for 8 weeks	None reported	Zung’s Depression and Anxiety scale, Self-Assessment Scale, SCL-90, BES	(52)
Xie et al. (2021), China	PCT	7 days	N = 13 I = 13	N = 30.0 ± 4.9	M = 6 F = 7	Healthy	Influence on serum biochemical markers and gut microbiota	1 g of HE powder 3 times a day	None reported	Chao1, ACE, Simpson, and Shannon indices	(21)
Li et al. (2020) Taiwan	PCT	365 days	N = 41 I = 20 C = 21	I = 74.3 ± 7.15 C = 77.05 ± 8.2	M = 17 F = 24	Patients	Prevention of early Alzheimer’s Disease	3 capsules containing 5 mg/g erinacine, an active ingredient, per day	Abdominal discomfort, nausea, and skin rash	NPI, CASI, MMSE, IADL	(19)
Docherty et al. (2023), United Kingdom	PCT	28 days	N = 43 I = 22 C = 21	N = 26.35 ± 6.12	M = 20 F = 23	Healthy	Improving cognitive function	Three capsules daily containing HE mushroom = 600 mg daily	None reported	COMPASS, S-VAS, VAMS, PSS	(20)
Grozier et al. (2022), United States of America	Cohort study	28 days	N = 24 I = 12 C = 12	I = 22.3 ± 3.2 C = 21.8 ± 2.7	M = 10 F = 14	Healthy	Impact on metabolic flexibility and cognition	10 g of HE in 2 muffins per day	None reported	Dual-task challenges consisting of a Stroop Word Challenge with a Mental Arithmetic Challenge and GXT	(22)
Nakatsugawa et al. (2003), Japan	Case report	120 days	N = 1 I = 1	N = 63	M = 1 F = 0	Patient	Not mentioned	HE dry powder extract as a diet food	ARDS	N/A	(23)

**Table 2:** Study characteristics: (laboratory studies and computer analysis).

Study	Design	Cell type	Study Focus	Treatment	References
Tian et al. (2022), China	Laboratory Study	Human Fecal Microbiota	Positive effect on Fecal Microbiota	Three polysaccharides were fractionally precipitated from hot water-soluble HE extracts using 30, 50, and 70% ethanol.	(43)
Kim et al. (2011)	Laboratory Study	U937 human monocytic leukemia cells	Induced apoptosis of monocytic leukemia	Hot water microwaved 50% ethanol, acidic, and alkaline extracts of the fruitbody of HE at 500 mg ml	(25)
Tada et al. (2022), Japan	Laboratory Study	The human monocytic leukemia THP-1 cells	Anti-inflammatory activities by neutralizing lipopolysaccharide-induced pro-inflammatory cytokine production in human monocytes	Ethanol and hot water extracts from HE	(24)
Li et al. (2015), Korea	Laboratory Study	HL-60 human acute promyelocytic leukemia and HEL-299 lung fibroblast cells	Anticancer activities against human acute promyelocytic leukemia	Hericerin A, hericerin J, and five known compounds at IC50 concentrations, were administered into HL-60 cells	(8)
Li et al. (2014), United States of America	Laboratory Study	HepG2 and Huh-7 liver, HT-29 colon, and NCI-87 gastric cancer	Anticancer potential against human gastrointestinal cancers	HTJ5 and HTJ5A in 100 μL	(7)
Zan et al. (2015), China	Laboratory Study	SGC-7901 human gastric carcinoma cells	Inhibits gastric carcinoma via cell cycle arrest and apoptosis	Cell exposure to HEG5 for 1–2 days for absorption	(26)
Ellan et al. (2019), Malaysia	Laboratory Study	Human peripheral blood mononuclear	Anti-inflammatory effect on dengue-infected human monocytes	Hot aqueous extraction (HAE) method and aqueous soluble extract (ASE)	(56)
Lai et al. (2013), Malaysia	Laboratory Study	neuroblastoma-glioma NG108-15 and lung fibroblast MRC-5	Neurotrophic properties	HE aqueous extract: 0–1,000 μg/mL tested NGF: 5–100 ng/mL tested Oxidative stress inducer (H2O2/hydrogen peroxide): 100 μM	(57)
Zhuang et al. (2023), China	Laboratory Study	human fecal microbiota	Antioxidant and prebiotic activities	HEP A and HEP W polysaccharides dried and ground	(42)
Tamrakar et al. (2023), Japan	Laboratory Study	human astrocytoma (BNDF mRNA expression)	Neuroprotective properties	Lipase enzyme treatment of Hericenone C and Deacyl hericenone	(58)
Hou et al. (2020), China	Laboratory Study	colorectal cancer: HCT-116 and DLD1	Induction of apoptosis in human colorectal cancer via ROS regeneration	HE fruiting body extracted and dried	(59)
Mori et al. (2009), Japan	Laboratory Study	human astrocytoma	Nerve growth factor-inducing activity in human astrocytoma cells	HE ethanol extract	(17)
Kuo et al. (2017)	Laboratory Study	gastric cancer	Inhibition of gastric cancer cell viability and invasiveness	HE ethanol extract isolating erinacine A	(36)
Chen et al. (2015), China	Laboratory Study	MCF-7 breast cancer and HeLa	Antioxidant and antiproliferation activities	Water extraction of HE polypeptide	(9)
Wang et al. (2019), Taiwan	Laboratory Study	BV2 microglial	Anti-inflammatory effect on microglial cells treated with LPS	Erinacine c through HE ethanol extraction	(60)
Sutthibutpong et al. (2024), Thailand	Computer Analysis	N/A	Identifying potential acetylcholinesterase inhibitors	Molecular docking to assess AChE inhibiting potential	(27)

#### 3.1.1 Randomized controlled trials

Three of the five RCTs reported in this systematic review were conducted in Japan. Four out of five RCTs involved healthy individuals, with all trials reporting benefits and minimal side effects. The effects of HE supplements were assessed for a wide range of conditions, including dementia, menopause, depression, anxiety, and mild cognitive impairment (17).

Evidence from one of the RCTs suggested improved cognitive function assessed through MMSE scores (30 in pre-treatment versus 29.19 in post-treatment) and improved Benton visual retention scores (7.13 in pre-treatment versus 6.88 in post-treatment). However, no differences in S-PA scores were observed between the control and intervention groups (1). A greater increase in MMSE scores was observed in a PCT assessing the effects of HE supplements on patients with early Alzheimer’s disease (23.2 in pre-treatment vs. 21.75 post-treatment). This improvement may be attributed to the increased dosage of HE supplements (17). Although HE is more commonly used to alleviate symptoms of cognitive impairment, it is also being considered for conditions such as menopause, irritation, and depression. One study reported a significant decrease in CES-D scores (MD: 3.6 and 2.5 in the intervention and placebo groups, respectively), as well as in KMI and ICI scores in HE groups compared to control (MD: 5.3 and 6 in the intervention and placebo groups, respectively). No difference was observed between the two groups for PSQI scores (5).

Although uncommonly documented, one RCT found that HE supplementation enhanced mood disorders characterized by depression and anxiety in obese patients, along with improving nocturnal rest quality. It elevated circulating pro-BDNF levels without notable changes in BDNF levels. In the long term, participants reported feeling less depressed and anxious following HE supplementation. The study also found that HE helped enhance working memory, complex attention, and reaction time immediately after ingestion. Additionally, the antioxidant effects of HE may provide additional health benefits for cognition with chronic use. No significant improvements in VAS mood (MD: 0.3), VAS mental clarity (MD: 0.2), or SHS general happiness (MD: 0.1) were recorded (18).

Though not often observed, some side effects of HE supplementation include headaches (18), stomach discomfort, diarrhea (17), and epimenorrhea (5).

#### 3.1.2 Pilot clinical studies

A year-long study in Taiwan provided robust evidence for the effects of HE supplementation in patients with mild AD. The placebo group exhibited a significant decline in the Cognitive Abilities Screening Instrument score (MD: 3.6), while the HE group demonstrated a significant improvement in the MMSE score (MD: 1.45). Furthermore, a notable difference in IADL scores was observed between the two groups (MD: 0.71 in the intervention group and 0.35 in the placebo group) (19).

Another pilot study reported that HE supplementation may improve performance speed and have potential stress-reducing effects; the investigation revealed that a single HE dosage resulted in quicker performance on the Stroop test 60 min after delivery. Furthermore, a decrease in subjective stress was seen after 28 days of supplementation (20).

Supplements increase the diversity of gut microbiota and short-chain fatty acids (SCFAs). They also lead to lower levels of alkaline phosphatase (ALP), uric acid (UA), creatinine (CREA), and low-density lipoprotein (LDL), along with the downregulation of pathobionts such as *Streptococcus thermophilus and Bacteroides caccae* (21).

#### 3.1.3 Cohort studies

One cohort study investigating the impact of HE supplementation on markers of metabolic flexibility or cognition was included in this systematic review. A total of 24 healthy adults participated in a placebo-controlled, single-blind, parallel longitudinal study to examine the effects of ingesting 10 g of HE daily for 4 weeks. The participants completed a fatiguing graded exercise test on a cycle ergometer to analyze substrate oxidation rates and markers of cardiorespiratory fitness. Subsequently, two dual-task challenges—the Stroop Color Word Test combined with a mental arithmetic task and the Y-Balance Test—were conducted to evaluate markers of cognition in the pre- and post-fatigue states. The study concluded that no significant effects were observed for any dependent variables. Therefore, the study concluded that the ingestion of HE did not affect metabolic flexibility or cognition (22).

#### 3.1.4 Case report

Our data screening identified one case report—interestingly, one of the rare cases wherein a side effect of potentially fatal severity was reported. It describes the case of a 63-year-old Japanese man with mild, untreated diabetes mellitus (DM) who had regularly been taking HE supplements from December 2001 until his emergency hospital admission for acute respiratory distress syndrome (ARDS). He presented with low-grade fever, hemosputum, cough, and exertional dyspnea; clinical findings revealed diffuse infiltration in both lungs (23).

A causal relationship between HE supplementation and ARDS was discussed, based on HE’s role in promoting NGF synthesis and producing immunomodulatory effects. A lymphocyte reaction test with the extract yielded significantly positive results, potentially strengthening the evidence. The report concluded that HE contains a compound capable of triggering an allergic reaction (23).

#### 3.1.5 Lab studies

Laboratory studies were primarily conducted in East Asian countries, including China (*N* = 5), Japan (*N* = 3), Korea (*N* = 1), and Taiwan (*N* = 1). The majority (*N* = 8) focused on HE effects on cancer cells, with all studies reporting benefits. Cancer-related studies mainly targeted leukemia and gastric cancer. Erinacine A significantly reduced cell proliferation and induced apoptosis in HL-60 cells (8). Other studies reported similar findings of decreased inflammatory responses in human monocytes caused by lipopolysaccharides (24), and extracts of HE induced NO production in macrophage cells, suppressing the proliferation of U937 and inducing apoptosis (25).

HE’s effect on gastric cancer cells shows that HEG-5 significantly affects apoptosis, cell proliferation, and the cell cycle of SGC-7901 by promoting cell cycle arrest at the S phase (26). However, there are also reports of HE extracts inducing *in vitro* cytotoxicity against gastrointestinal cancers (7).

#### 3.1.6 Computer analysis

One computer analysis study was identified during our screening. A combination of machine learning and structural modeling was used to identify compounds from HE with AChE-inhibiting activities (27).

A deep learning neural network was developed to rapidly screen compounds for their potential to inhibit AChE activity. Deep learning models were also applied to screen compounds in the BACMUSHBASE database (28). Five promising compounds from HE were identified, among which erinacine A and hericenone B were selected based on further analysis (27).

Subsequently, molecular dynamics simulations and MM/PBSA free energy calculations for erinacine A and hericenone B were compared with those of approved drug molecules. Further analysis revealed that these compounds exhibited binding energy profiles similar to those of donepezil and galantamine. Similarities were also observed in their binding mechanisms, suggesting that erinacine A and hericenone B may have the potential to serve as alternative AChE-targeting drugs. The study concluded that both compounds are suitable candidates for further research and development as AD medications (27).

### 3.2 Toxicology report

Table 3 summarizes the dosage- and toxicity-related data, including variations in dosage forms, concentrations, administration durations, reported adverse effects, toxicology findings, compliance rates, and analytical tools used across all 25 studies included in our systematic review, excluding the computer analysis due to insufficient data. The compiled data provide an overview of the safety profile, cytotoxicity evaluations, and experimental parameters used to assess HE’s therapeutic potential.

**Table 3:** Dosage and toxicology characteristics.

Study	Dosage form	Dosage concentration	Dosage duration	Adverse side effects	Toxicology	Compliance	Tools used (assays)	References
Saitsu et al. (2019)	Supplement contained fruiting powder	4/day (3.2 g daily)	12 weeks	None reported	None reported	High Compliance	As mentioned in Table 1	(1)
Nagano et al. (2010)	Cookies containing powdered fruiting bodies	4/day (2.0 g HE powder/day)	4 weeks	One participant reported epimenorrhea but was excluded from the sample, not attributed to HE	None reported	High Compliance		(5)
Mori et al. (2009)	Tablets contained 96% Yamabushitake dry powder	4 tablets 3/day (3.0 g / day)	Intake: 16 Follow-Up: 4	Mild stomach discomfort & diarrhea in 7 Yamabushitake vs. 6 placebo; no treatment needed	None reported	High Compliance		(17)
La Monica et al. (2022)	Capsule containing dried powder of the fruiting body	250 mg 4/day (1.0 g/day)	16 weeks	Mild stomach discomfort	None reported	None reported		(18)
Vigna et al. (2019)	Capsule containing mycelium and fruiting body extract	400 mg HE mycelium and 100 mg HE fruiting body extract per capsule 3/day (1.5 g/day)	8 weeks	None reported	None reported	High Compliance		(52)
Xie et al. (2021)	Dry Powder	1 g 3/day (3.0 g/day)	1 weeks	None reported	None reported	None reported		(21)
Li et al. (2020)	Dry powder containing fruiting bodies	350 mg/capsule containing 5 mg/g erinacine A 3/day	49 weeks	Nausea, abdominal discomfort, and nausea	None reported	Average compliance: 14.3% dropout		(19)
Docherty et al. (2023)	Capsule	600 mg 3 /day 1.8 g/day	4 weeks	None reported	None reported	High Compliance		(20)
Grozier et al. (2022)	Muffins containing HE fruiting body powder	2 muffins/day (10 g/day)	4 weeks	None reported	None reported	High Compliance		(22)
Nakatsugawa et al. (2003)	Dry powder extract of HE	Not specified (commercial quantities)	16 weeks	Acute Respiratory Distress Syndrome (ARDS)	Elevated serum SP-A and SP-D indicate lung injury; strong lymphocyte proliferation	Daily use		(23)
Tada et al. (2022)	Ethanol and hot water extracts from HE	N/A	24 h	N/A	No direct cytotoxicity to human monocytic cells	N/A	Enzyme-linked immunosorbent assay (ELISA)	(24)
Li et al. (2015)	Hericerin A at IC50 concentrations was administered into HL-60 cells	0.01–100 μM	72 h	N/A	Hericerin A (IC₅₀ = 3.06 μM) showed potent, selective cytotoxicity against HL-60 leukemia cells with minimal toxicity to normal HEL-299 cells (>50 μM)	N/A	MTT cell viability; Flow cytometry (PI, sub-G1 population); Hoechst 33342 nuclear morphology, Western blot	(8)
Li et al. (2014)	HTJ5 and HTJ5A in 100 μL	0.156–20.0 mg/mL	72 h	N/A	HTJ5 and HTJ5A exhibited concentration-dependent cytotoxicity *in vitro* against liver cancer HepG2 and Huh-7, colon cancer HT-29, and gastric cancer NCI-87 cells	N/A	MTT assay	(7)
Zan et al. (2015)	Hericium erinaceus polysaccharide-protein HEG-5	10–200 μg/mL	24–48 h	N/A	Dose-dependent reduction in cell viability (up to 93.4% inhibition at 200 μg/mL), induced apoptosis, and cell cycle arrest	N/A	MTT assay, Annexin V-FITC and PI Double Staining, Cell Cycle Distribution Assay, Comet Assay, qRT-PCR, and Western blot analysis	(26)
Ellan et al. (2019)	Hot aqueous extract and aqueous-soluble fraction were separated from the ethanol extract	313–1,500 μg/mL	48 h	N/A	Non-cytotoxic up to 1,500 μg/mL; no significant inhibition of cytokines at non-cytotoxic doses	N/A	MTT Assay, ELISA Cytokine Assay, and IC₅₀ analysis	(56)
Lai et al. (2013)	Aqueous extract (hot water-decocted and freeze-dried)	1–1,000 μg/mL	24–48 h	N/A	No significant cytotoxicity at the tested doses; protective effects noted under oxidative stress	N/A	MTT Assay, Trypan Blue Assay, TUNEL Assay	(57)
Zhuang et al. (2022)	HEP-A and HEP-W	1–3,000 μg/mL	N/A	N/A	No cytotoxic effects were reported at tested concentrations	N/A	DPPH, ABTS, radical scavenging assay, Hydroxyl radical-scavenging assay, Simulated saliva gastrointestinal digestion, Human fecal fermentation model, Ion chromatography, HPGFC	(42)
Tamrakar et al. (2023)	Ethanol extract of hericenone C and its deacylated derivative (deacyl hericenone)	1.6–12.5 μg/mL	24 h	N/A	No indications of cytotoxicity at tested doses; deacyl hericenone showed enhanced protective effects over the parent compound in the oxidative stress model	N/A	LC–QTOF–MS, ^1^H-NMR, BDNF mRNA expression assays, Oxidative stress cell viability assay	(58)
Hou et al. (2020)	HE fruiting body polysaccharides	Not specified	12–120 h	N/A	Selective cytotoxicity to cancer cells via the reactive oxygen species-mediated caspase-9 pathway and intrinsic apoptotic pathway	N/A	Cell Viability Assay, Reactive Oxygen Species Measurement Assay, Mitochondrial Membrane Potential Assay, Western Blot	(59)
Mori et al. (2008)	Ethanol extract of HE fruiting bodies	50–250 μg/mL	3–168 h	N/A	No cytotoxicity evident at tested doses	N/A	MTT Assay, RT-PCR, Enzyme Immunoassay of NGF	(50)
Kuo et al. (2017)	Erinacine A	1–10 μM	24 h	N/A	Dose-dependent cytotoxicity and apoptosis	N/A	MTT Assay, Boyden Chamber Assay, Annexin V–FITC/ Propidium Iodide staining, Proteomics Analysis, Western Blot	(36)
Chen et al. (2015)	Water-extracted and alcohol-precipitated polysaccharides from HE	50–200 μg/mL	24 h	N/A	Dose-dependent inhibition of cancer cell proliferation	N/A	MTT Assay, DPPḤ Radical-Scavenging Assay, Hydroxyl Radical-Scavenging Assay	(9)
Wang et al. (2019)	Erinacine C	0.1–10 μM	25 h	N/A	No cytotoxicity observed at 0.1–2.5 μM	N/A	Cell Growth Analysis (Hemocytometer), Nitric Oxide Assay, ELISA, Western Blot	(60)
Tian et al. (2022)	Three polysaccharides from hot water-soluble extracts of HE	8 mg/mL	24 h	N/A	No direct report of toxicity or adverse effects	N/A	No toxicology assays performed	(43)
Kim et al. (2011)	Hot water, ethanol, acidic, and alkaline extracts of the fruiting body of HE	500 μg/mL	2–48 h	N/A	HWE and MWE decreased the viability of U937 cells; ACE and AKE (controls) were not cytotoxic	N/A	MTT assay to assess cell viability, Caspase activity assays (caspase-3, −8, −9), Mitochondrial membrane potential (MMP) assay,	(25)

## 4 Discussion

This systematic review includes the results of five RCTs, three PCTs, fifteen laboratory studies, one cohort study, one case report, and one computer analysis. Ample evidence from existing literature suggests a link between the neurotrophin family of proteins, such as NGF and BDNF, and diterpenoids (erinacine A, B, and C) extracted from the mycelia of HE, which exhibit strong neurotrophin-stimulating activity. Findings from several spectral and enzymatic laboratory studies further support the potential of HE as a beneficial supplement for a wide range of conditions, from age-related neurodegenerative disorders, including dementia, AD, and PD, to mood-related disorders such as anxiety, depression, insomnia, and binge eating. HE plays a vital role in enhancing gut microbiota by increasing the abundance of SCFAs-producing bacteria and reducing harmful bacteria. Moreover, its bioactive agents help maintain the integrity of the gut–brain axis by minimizing inflammation and mitigating age-related cellular changes. Compounds from HE, such as erinacine A and HEG-5, possess potent anticancer properties by inducing apoptosis and suppressing cancer cell growth. They act on essential pathways such as caspase activation, mitochondrial-mediated apoptosis, and PI3K/AKT signaling while suppressing anti-apoptotic proteins such as Bcl-2. In our review, although rare, reported side effects of HE supplementation included stomach discomfort, diarrhea (17, 19), headaches (18), and epimenorrhea (5). One case report from Japan documented acute respiratory distress syndrome in a 63-year-old male following prolonged use of HE.

Previously, only three systematic reviews had been conducted on the uses of HE. One concluded a possible role for herbal remedies in delaying the progression of spinocerebellar ataxia type 3 (29), while another similar study reported the effective medicinal use of HE for hereditary ataxia (30). Compared to other nutritional supplements, HE mycelia enhanced cognitive, functional, and quality of life in individuals assessed for malnutrition and AD (31). Given the limited number of reviews, this systematic review focuses singularly on the effects of HE to provide a more comprehensive analysis of its potential as a medicinal mushroom.

### 4.1 Neurotrophins and neurodegenerative disorders

#### 4.1.1 Role of HE in neurotrophin signaling and cognitive function

In our review, multiple studies showed that HE provides beneficial effects on cognitive function and neurotrophic regulation (1, 17). These cognitive benefits are attributed to its capacity to stimulate NGF synthesis and support neuroplasticity (20). Neurotrophins are essential for the survival, adaptation, and differentiation of neurons and are primarily associated with the central nervous system. Survival signals are mediated through the activation of the high-affinity receptor tropomyosin receptor kinase A (TrkA), while cell death is induced via p75NTR binding. Erinacines A, B, and C are diterpenoids isolated from HE-cultured mycelia, and spectral and enzymatic analysis have shown that they exhibit strong NGF-stimulating activity (2).

One study in our review reported that HE supplementation increased circulating pro-BDNF concentrations without a corresponding change in mature BDNF levels (28). BDNF plays a central role in learning, memory consolidation, and synaptic plasticity (29). Homozygous mutation of the BDNF gene has been associated with degeneration in sensory and vestibular pathways, leading to coordination deficits (31). Overexpression of TrkB enhances memory, while underexpression leads to impaired spatial memory. BDNF is also associated with aging, and some recent findings suggest that age-related physiological and pathological changes in the CNS can be altered by either stimulating receptor expression or by varying BDNF administration. Additionally, BDNF supports the survival and function of several neuronal populations, including mesencephalic dopaminergic, septal cholinergic, and striatal GABAergic neurons (30).

Neuronal function and survival depend on balanced levels of reactive oxygen species (ROS) and reactive nitrogen species (RNS) to maintain synaptic plasticity. Excessive ROS/RNS levels are known to play a detrimental role in the pathogenesis of neurodegenerative disorders. Substantial evidence indicates that neurodegenerative diseases such as Alzheimer’s disease (AD), Parkinson’s disease (PD), and Huntington’s disease (HD) are characterized by increased levels of oxidation markers and decreased antioxidant ability, leading to excitotoxicity, mitochondrial dysfunction, and apoptosis. The role of BDNF is particularly significant in inhibiting neuronal death caused by oxidative stress–mediated degeneration, ischemia, and amyloid-*β* toxicity (31, 32).

#### 4.1.2 Clinical and preclinical findings

Across the studies included in our review, administration of HE was generally associated with beneficial signals on cognitive outcomes and neurotrophic markers, although the evidence base remains heterogeneous and limited in size. One such study (1) reported increased MMSE scores when comparing pre- and post-intervention assessments, indicating improved global cognition (MD: 0.81). Consistent directional effects were observed across randomized controlled trials and experimental models, including an earlier study by Mori et al. (17), which also found that HE is effective in improving mild cognitive impairment.

In a similar study evaluating both acute and 28-day chronic HE administration (1.8 g/day), a single dose produced a statistically significant improvement in Stroop task speed (*p* = 0.005). With continued daily intake over 28 days, investigators observed a trend toward reduced stress (*p* = 0.051) but did not detect significant gains in cognition or memory, underscoring the need for longer-duration trials (20).

### 4.2 Cancer and apoptosis

All cancer-related studies included in this review were *in vitro* laboratory studies, with three focusing explicitly on leukemia (8, 24, 25) and two assessing gastric cancer (26, 36), while other studies employed a holistic approach in evaluating more than one cancer type or process. A key finding among these studies was that the bioactive compounds of HE, such as erinacine A, hericerin A, and HEG-5, contribute to the induction of apoptosis in several cancer cell lines.

Previous research demonstrates that erinacine A, when isolated from the HE mycelium, inhibits the viability and invasiveness of gastric cancer cells, including MKN28 and TSGH 9201 cells. They also found that erinacine A induces the accumulation of TSGH 9201 cells in a time-dependent manner, with the induction of apoptosis.

#### 4.2.1 Molecular mechanisms of apoptosis

Apoptosis can occur through either an extrinsic or death receptor-mediated pathway, or an intrinsic, mitochondrial-mediated pathway. The triggering event of the extrinsic pathway is when death ligands such as Fas ligand, tumor necrosis factor-related apoptosis-inducing ligand, or tumor necrosis factor receptor 1 bind to their cell surface death receptors, triggering the formation of the death-inducing signaling complex, leading to the activation of caspase-8. This activates downstream effector caspases such as caspase-3 to execute apoptosis. Additionally, the intrinsic pathway is activated by intracellular stress signals, such as ROS or DNA damage. These stress signals cause mitochondrial outer membrane permeabilization, leading to the release of cytochrome c into the cytosol (37). Cytochrome c then binds with Apaf-1 and procaspase-9 to form a complex called the apoptosome. This results in the activation of caspase-9, which subsequently activates caspase-3 to carry out apoptosis. The role of pro-apoptotic proteins Bax and Bad, and anti-apoptotic proteins such as Bcl-2 and Bcl-XL, is to regulate this pathway by balancing mitochondrial membrane permeability, either by allowing or preventing the release of cytochrome c (38).

The phosphoinositide 3-kinase/protein kinase B signaling pathway (PI3K/AKT) is a key component of numerous biological processes, including cell growth and proliferation, survival and apoptosis, metabolic regulation, angiogenesis, as well as adipogenesis and adipocyte differentiation. When activated, the pathway promotes the activity of anti-apoptotic proteins such as B-cell lymphoma 2 and B-cell lymphoma extra-large, while suppressing pro-apoptotic molecules. This stabilizes the mitochondrial membrane and prevents cell death. In cancer, the continuous activation of the PI3K/AKT pathway supports uncontrolled proliferation and resistance to treatment by blocking key steps in cellular apoptosis (39, 40).

#### 4.2.2 Clinical and preclinical evidence

Experimental data now clearly demonstrate that HE compounds, such as erinacine A, induce apoptosis through the intrinsic mitochondrial pathway. These mechanisms include the upregulation of pro-apoptotic proteins such as Bax, the downregulation of anti-apoptotic proteins such as Bcl-2 and Bcl-xL, and the release of cytochrome c, alongside increased TRAIL expression and ROS generation. The loss of mitochondrial membrane potential and the activation of cleaved caspase 9 and caspase 3 are all key characteristics of mitochondrial apoptosis. A proteomic analysis of human gastric cancer cells treated with erinacine A revealed notable shifts in protein expression, marked by the upregulation of tumor suppressors 14-3-3σ (1433S) and MTUS2, and a reduction in nucleophosmin (NPM). In addition to these findings, increased ROS production and the activation of the FAK/AKT/p70S6K and PAK1 signaling pathways suggest a mechanism that drives apoptosis and reduces the invasiveness of gastric cancer cells (36).

A study assessing the induction of apoptosis by erinacine A in human colorectal cancer cells demonstrated that erinacine A elevates the expression of the principal death receptor components of the extrinsic apoptotic pathway: tumor necrosis factor receptor-1 (TNFR1), Fas (CD95), and Fas ligand (FasL). Their coordinated upregulation promotes the assembly of death-inducing signaling complexes at the plasma membrane, thereby activating the initiator caspase-8 via proteolytic cleavage.

Activated caspase-8 then propagates the downstream caspase cascade that executes apoptosis. In parallel, erinacine A enhances intracellular signaling through c-Jun N-terminal kinase (JNK), the transcriptional co-activator and histone acetyltransferase p300, and the NFκB (p50) axis. JNK phosphorylation modulates transcription factor activity; p300 mediates chromatin relaxation through histone acetylation to increase promoter accessibility; and NFκB p50 contributes to the transcriptional activation of apoptosis-related target genes (41).

Several studies included in this review corroborate these findings, showing that treatment with erinacine A results in the activation of TRAIL, caspase 8, caspase 9, and caspase 3, as well as the release of cytochrome c, whereas there is a decrease in the cellular levels of Bcl-2 and Bcl-XL in erinacine A-induced apoptosis (36).

Another study in this review found that hericerin A effectively inhibited the growth of HL 60 human acute promyelocytic leukemia cells. This growth inhibition was predominantly caused by the activation of apoptosis, as demonstrated by cellular alterations such as chromatin condensation, the development of apoptotic bodies, and an increase in sub-G1 cell numbers. In addition, these compounds were shown to upregulate the pro-apoptotic protein Bax while downregulating the anti-apoptotic protein Bcl-2, resulting in caspase-3 activation and subsequent poly (ADP-ribose) polymerase cleavage, as observed in previous studies.

Furthermore, its pro-apoptotic activity was connected to the inhibition of the PI3K/AKT signaling pathway, with lower levels of phosphorylated AKT and the oncogenic transcription factor c-myc. Notably, these compounds showed minimal cytotoxicity against normal HEL-299 fibroblasts, indicating their potential as selective anticancer agents (8). HEG-5, a compound found within *Hericium erinaceus*, significantly inhibited the proliferation and colony formation of 19 SGC-7901 cells by inducing apoptosis and causing cell cycle arrest at the S phase. RT-PCR and Western blot analysis suggested that HEG-5 could decrease the expression of Bcl2, PI3K, and AKT1 (21) while increasing the expression of Caspase-8, Caspase-3, p53, CDK4, Bax, and Bad. These findings indicate that Caspase-8/−3-dependent, p53-dependent mitochondrial-mediated pathways (23), along with the PI3k/Akt signaling pathways, are involved in the molecular events leading to HEG-5–induced apoptosis and cell cycle arrest (26).

Despite the promising anticancer effects of HE’s bioactive compounds demonstrated *in vitro*, several limitations must be acknowledged. The majority of studies have been conducted using cancer cell lines under controlled laboratory conditions, and they do not account for the complexity of tumor biology, such as *in vivo* models, including tumor microenvironment interactions, immune responses, and pharmacokinetics. Furthermore, there is a lack of *in vivo* studies and clinical trials investigating the efficacy, bioavailability, metabolism, and potential toxicity of HE compounds such as erinacine A, hericerin, and HEG-5. Given the increased variability in experimental designs, there is a lack of generalizability of findings across studies. Additionally, although several signaling pathways have been implicated in HE-induced apoptosis, the precise molecular targets and their effects are not fully characterized. Moreover, while the selective cytotoxicity of HE compounds toward cancer cells over normal cells is encouraging, it requires further validation in physiologically relevant models to confirm therapeutic windows and minimize adverse effects.

### 4.3 Gut microbiota

#### 4.3.1 Microbiota modulation

HE is rich in a variety of active ingredients such as polysaccharides, diterpenes, pyranones, phenols, and sterols (42). Recently, polysaccharides have become the primary derivatives of HE and have become the subject of research. HEPs exert their effects on SCFA production through several mechanisms, primarily attributed to their high content of indigestible polysaccharides, especially *β*-glucans and heteropolysaccharides. These bioactive polysaccharides largely resist digestion in the upper gastrointestinal tract, reaching the colon intact and acting as fermentable substrates for beneficial gut microbiota (43, 44). Notably, HE polysaccharides stimulate SCFA-producing bacteria, enhancing the synthesis of SCFAs, particularly butyrate, acetate, and propionate (21, 43). Evidence from both animal models and *in vitro* studies demonstrates that fermentation of HEPs prompts an increase in the abundance of these beneficial genera, resulting in elevated levels of total and specific SCFAs in the gut.

SCFAs are carboxylic acids with fewer than six carbon atoms, primarily comprising acetate (C2), propionate (C3), and butyrate (C4). These key metabolites are produced by gut microbiota through the anaerobic fermentation of indigestible carbohydrates, including dietary fibers and polysaccharides, in the colon (45, 46). SCFAs exert systemic and local physiological effects via multiple mechanisms, including serving as a key energy source for colonocytes, supporting the intestinal barrier’s integrity by promoting tight junction proteins (e.g., claudin-1, zonula occludens-1) and mucins (e.g., Mucin 2), and modulating immune responses via both direct and receptor-mediated pathways (45). SCFAs function as important signaling molecules, particularly through the activation of G-protein-coupled receptors such as FFAR2 (GPR43), FFAR3 (GPR41), and HCA2 (GPR109A), which are expressed on diverse cell types, including immune cells, colonocytes, and neurons. Additionally, SCFAs, especially butyrate, influence gene expression by inhibiting histone deacetylases, thereby affecting anti-inflammatory processes and cellular differentiation (45, 47).

Furthermore, SCFAs support metabolic regulation by influencing gluconeogenesis and lipid metabolism, increasing the production of glucagon-like peptide 1, suppressing inflammatory processes, protecting against insulin resistance, and exhibiting anti-tumor activity. Deficiencies or imbalances in SCFA production have been associated with disorders such as inflammatory bowel disease, metabolic syndrome, and certain neuropsychiatric conditions, highlighting their essential role in maintaining gut and overall health (45, 46).

#### 4.3.2 Clinical and preclinical findings

Recent literature suggests that HE supplementation may influence the alpha diversity of gut microbiota and upregulate SCFA-producing bacteria in humans. The primary clinical evidence in this regard is derived from a single PCT conducted in China, which concluded that daily HE supplementation over seven days in healthy adults led to a significant increase in SCFA-producing genera, including *Kineothrix alysoides*, *Gemmiger formicilis*, *Fusicatenibacter saccharivorans*, *Eubacterium rectale*, and *Faecalibacterium prausnitzii*. Concurrently, potential opportunistic bacteria such as *Streptococcus thermophilus*, *Bacteroides caccae*, and *Romboutsia timonensis* were downregulated. The shift in microbiota diversity was associated with a favorable reduction in levels of alkaline phosphatase, low-density lipoprotein, uric acid, and creatinine after HE intervention (21).

Although promising, it should be noted that this was the only published human clinical study meeting our inclusion criteria during our comprehensive and systematic search that specifically examined the influence of HE supplementation on SCFA-producing gut bacteria. While this highlights a gap in current clinical literature, the existence of a single robust clinical study with clear and measurable outcomes provides valuable and direct evidence supporting this association.

These results are also corroborated by a number of *in vitro* and animal studies, which consistently support the findings regarding the effects of HE on gut microbiota and SCFA production. An *in vitro* study investigated 24 h of fermentation and found that HEPs stimulated total SCFA production (including acetate, propionate, and butyrate) and increased the abundance of SCFA-producing bacteria, namely *Bifidobacterium, Faecalibacterium, Blautia, Butyricicoccus, and Lactobacillus*. Furthermore, a notable decrease in the relative abundance of opportunistic pathogens, such as *Escherichia-Shigella, Klebsiella, and Enterobacter,* was observed (43). These studies indicate that the fermentation of HE *β*-glucans not only increases overall SCFA production but also promotes the proliferation of SCFAs-producing bacterial genera through microbial cross-feeding interactions (44). Therefore, HE β-glucans function as prebiotics, shaping both the composition and metabolic activity of the gut microbiota to favor enhanced SCFA synthesis. Animal studies further support these findings. In rats, supplementation with HEPs led to notable enrichment of beneficial gut bacteria and elevated levels of SCFAs, alongside improvements in intestinal barrier integrity and reductions in inflammation. Similarly, aged dogs fed an HE-enriched diet for sixteen weeks exhibited remarkable shifts in gut microbiota composition, with an increased prevalence of SCFA-producing *Bacteroidetes* (48). In mice, HEPs supplementation promoted the growth of SCFA-producing bacteria, namely *Clostridia UCG-014, Lachnospiraceae_NK4A136, and Eubacterium xylanophilum,* while decreasing the abundance of potentially harmful microbes, collectively supporting both gut and systemic health (49). Multiple studies have investigated the modulation of gut physiology by the central nervous system, also known as the gut-brain axis. This bidirectional system integrates neural, immune, endocrine, and metabolic signaling pathways to regulate numerous functions, such as motility, secretion, and nociception. HEPs foster the proliferation of beneficial gut microbiota, such as *Faecalibacterium, Bifidobacterium, and Blautia*, which modulate the synthesis of neuroactive compounds, neurotransmitters, and immune mediators, including serotonin and gamma-aminobutyric acid (GABA), that communicate directly with the central nervous system (21, 46).

HE contains neuroactive compounds, namely erinacines and hericenones, which promote the synthesis of neurotrophins such as NGF and BDNF, both essential for neural growth, synaptic plasticity, and overall neuronal health (50). By increasing the abundance of favorable microbiota, HE increases the production of microbial metabolites such as SCFAs, which contribute to an anti-inflammatory effect supporting both gut and brain health (46). SCFAs reach the brain either by crossing the blood–brain barrier or through neural signaling pathways such as the vagus nerve, influencing neuroinflammation and neural circuits involved in mood, cognition, and neuroprotection (51). This allows SCFAs to interact with microglia, astrocytes, and neurons, mediating their effects through G-protein-coupled receptors (FFAR2/FFAR3), epigenetic modifications (including inhibition of histone deacetylases), and modulation of BDNF in brain regions involved in emotion and cognition. Furthermore, the shifts in microbiome composition induced by HE have demonstrated reduced gut permeability, thereby limiting the translocation of bacterial components such as lipopolysaccharides into the bloodstream and subsequently diminishing both systemic and neuroinflammation. Elevated lipopolysaccharide levels influence neuroinflammatory cascades, which are strongly associated with depression, anxiety, and neurodegenerative disorders (46, 51). Beyond these immunomodulatory effects, preclinical trials indicate that HE and its metabolites can activate signaling pathways such as ERK/CREB in hippocampal neurons, increasing neurotrophic factor expression and supporting neurogenesis (50). Collectively, these findings suggest that HE can exert a multifaceted influence on the gut-brain axis, enhancing microbiota composition, reducing inflammation, and directly modulating neurotrophic and neurotransmitter systems.

### 4.4 Mental health

#### 4.4.1 Influence on mood and emotional health

HE’s bioactive compounds, particularly hericenones and erinacines, can cross the blood–brain barrier and enhance the production of neurotrophic factors such as NGF, BDNF, and pro-BDNF. NGF and BDNF are vital for synaptic plasticity, neurogenesis, and overall neuronal survival, whereas pro-BDNF activates apoptotic pathways in neurons and glial cells. An imbalance or defect in these neurotrophins could result in cognitive impairment, psychiatric disorders, and anxious behavior (52). A key mechanism involves the hypothalamic–pituitary–adrenal (HPA) axis, a critical neuroendocrine system that modulates stress responses. Animal studies suggest that supplementation with HE can normalize the activity of the HPA axis by attenuating stress-induced elevations in corticosterone, thereby reducing both physiological and behavioral indicators of chronic stress. Furthermore, evidence suggests that HE supplementation modulates the gut-brain axis by promoting the growth of beneficial gut bacteria that produce anti-inflammatory and neuroactive metabolites, such as SCFAs, which influence brain function. Emerging research further implicates HE in the regulation of neurotransmitter synthesis and signaling, including serotonin and GABA, which are integral to emotional stability and mood regulation.

#### 4.4.2 Clinical and preclinical findings

Several clinical and preclinical studies have demonstrated that HE supplementation leads to reductions in symptoms associated with depression, anxiety, binge eating, and sleep disorders, as demonstrated by significant changes in standardized scales such as CES-D and KMI (5, 52). For example, an 8-week supplementation trial involving overweight and obese adults revealed significant decreases in depression and anxiety scores, measured by Zung’s depression and anxiety scales. Increases in pro-BDNF/BDNF ratios were also observed within this study, suggesting a link between neurotrophic support and psychological outcomes.

Additionally, a double-blind, placebo-controlled trial investigated the potential of daily HE consumption in individuals diagnosed with AD. The study utilized a comprehensive list of neuropsychological assessments, including the Neuropsychiatric Inventory (NPI) for behavioral symptoms, the Cognitive Abilities Screening Instrument (CASI) to evaluate multiple cognitive domains, the MMSE for global cognitive function, and the Instrumental Activities of Daily Living (IADL) to understand functional autonomy. The intervention group demonstrated notable improvements in NPI scores, reflecting a reduction in neuropsychiatric symptoms such as agitation, depression, and anxiety. HE consumption also resulted in an increase in CASI scores, indicating enhanced attention, memory, language, and visual construction abilities.

Furthermore, a significant increase in the MMSE scores of the intervention group was noted, suggesting either maintained or improved cognitive status compared to the placebo group, potentially mitigating cognitive decline. Lastly, IADL scores were also greater in the HE group, representing a lower dependence level and participants’ improved ability to perform everyday tasks independently. The findings provide preliminary but encouraging evidence of the potential of HE to exert beneficial effects on both behavioral and cognitive symptoms in individuals with mild AD (19).

Another clinical study examined the impact of HE on psychological well-being and menopausal symptoms in a randomized cohort. Participants were assessed using several validated tools, including the KMI, CES-D, PSQI, and ICI. Participants from the HE groups obtained significantly lower scores on the CES-D, suggesting a decrease in depressive symptoms. Moreover, upon HE consumption, scores on the ICI showed a significant decline, with the most remarkable advances being in symptoms such as “palpitation,” “insensitive,” “irritating,” “anxiety,” and “concentration” (5), indicating that HE can produce a meaningful reduction in symptoms associated with depression and anxiety through the promotion of hippocampal neurogenesis, which has been proven to be correlated with behavioral and emotional improvements. Additionally, HE consumption leads to the modulation of serotonin receptors, which contributes to the anxiolytic and antidepressant effects observed (53).

### 4.5 Limitations

This systematic review is the only comprehensive review on PubMed that investigates the benefits and side effects of *Hericium erinaceus*. Additionally, this review includes studies conducted in numerous countries across various continents, strengthening the findings’ external validity.

A key limitation of this systematic review is the inability to perform a meta-analysis, as detailed in the results section. This was due to incomplete and unclear reporting in the studies included, such as a lack of separate first-period data in crossover trials and discrepancies in statistical values like standard deviations. Future studies should prioritize transparent reporting of first-period results in crossover trials to enable reliable meta-analytic synthesis and strengthen the robustness of systematic review conclusions. Another limitation is the use of a single database (PubMed), which may introduce selection bias. While other databases, such as Embase and Scopus, were considered, given that the data retrieved from them overlapped with the data already retrieved from PubMed as duplicated articles, the inclusion of other databases was unlikely to enhance the breadth or quality of the literature reviewed significantly. Since PubMed already aggregates a broad range of medical journals, the addition of another database was not considered necessary, given the extensive coverage provided by PubMed.

The study acknowledged that a type II error may have occurred because no power analysis was conducted before the intervention, leading to no significant differences between the groups, and that the dosage used may not have been sufficient to induce metabolic or cognitive changes (22).

The case report documenting the fatal side effects of HE was the first and only of its kind to be recorded. Japan has seen an increase in HE being used as a dietary supplement, and China has used it in herbal medicine; therefore, the cause of ARDS cannot be entirely attributed to HE without further investigation (23).

HE’s effectiveness as a dietary supplement has scarcely been tested. We found more scientific evidence exploring HE’s capacity as a therapeutic intervention in mice for improving a variety of conditions, including gut microbiota–brain axis (54), post-injury axon regeneration (53), anti-obesity activity (55), gastric mucosal protection (53), and lipid metabolism (55). However, there is still a lack of substantial evidence to support the capacity of HE as a therapeutic supplement for humans. Existing randomized controlled trials mainly focus on HE’s role in improving cognition, dementia, anxiety, and depression (1, 5, 17, 18, 52).

## 5 Conclusion

HE, as a dietary supplement, shows limited effectiveness in clinical trials and is primarily used for temporary improvement in cognitive function and mental clarity. Although erinacines and hericenones derived from its mycelium have shown promising stimulation of NGF, the majority of studies report only limited improvement in neural functional enhancement. The anti-tumor effects of HE are a relatively new area of research and are currently limited to *in vitro* studies; its mode of action and anti-apoptotic activity have not yet been clearly established, although this remains a promising area of research. Only a few studies have explored the effects of HE on the gut–brain axis, mood disorders, and sleep disorders. However, the existing studies demonstrate strong potential in reducing symptoms of mood disorders and sleep disorders and in positively modulating the gut–brain axis, thereby supporting immune function and enhancing cognitive performance.

Despite promising findings from randomized controlled trials, pilot studies, and laboratory investigations, current research is limited by sample sizes, short study durations, and heterogeneity across studies. Future research should involve larger and more diverse populations—considering factors such as age, gender, and ethnicity—when exploring various neurodegenerative and mood disorders to establish a safety profile accurately. Additionally, a sustainable agricultural approach should be developed to support the conservation of HE, given its growing demand.

## Data Availability

The original contributions presented in the study are included in the article/Supplementary material, further inquiries can be directed to the corresponding authors.

## Glossary

HE: Hericium Erinaceus
NGF: Nerve Growth Factor
BDNF: Brain-Derived Neurotrophic Factor
SCFAs: Short-Chain Fatty Acids
AD: Alzheimer’s Disease
PD: Parkinson’s Disease
MMSE: Mini-Mental State Examination
CES-D: Center for Epidemiological Studies-Depression
KMI: Kuperman Menopausal Index
PSQI: Pittsburgh Sleep Quality Index
ICI: Indefinite Complaints Index
PCT: Pilot Clinical Trial
RCT: Randomized Controlled Trial
AChE: Acetylcholinesterase
HEP: Hericium Erinaceus Polysaccharide
TrkA: Tropomyosin receptor kinase A
ROS: Reactive Oxygen Species
RNS: Reactive Nitrogen Species
PI3K/AKT: Phosphoinositide 3-kinase/AKT serine–threonine kinase pathway
HDS-R: Hasegawa’s Dementia Scale-Revised
VAS: Visual Analogue Scale
SHS: Self-Rated Health Scale
NPI: Neuropsychiatric Inventory
CASI: Cognitive Abilities Screening Instrument
IADL: Instrumental Activities of Daily Living
COMPASS: Composite Autonomic Symptom Score
VAMS: Visual Analogue Mood Scale
PSS: Perceived Stress Scale
GXT: Graded Exercise Test
