# Supplementary material for: Benefits, side effects, and uses of Hericium erinaceus as a supplement: a systematic review
Source: Front Nutr. 2025 Sep 1;12:1641246. doi: 10.3389/fnut.2025.1641246 (PMC12434001; doi:10.3389/fnut.2025.1641246)
Supplement: Supplementary file 1 [file Table_1.DOCX]

**Supplementary Material**

1. **APPENDIX:**

The Boolean search strategy string is as follows (("Hericium Erinaceus"[Title/Abstract]) OR ("Lion's mane"[Title/Abstract]) OR ("monkey* head mushroom"[Title/Abstract]) OR (yamabushitake[Title/Abstract])) AND ((benefit[Title/Abstract]) OR (advantage[Title/Abstract]) OR ("mental health"[Title/Abstract]) OR (Alzheimer*[Title/Abstract]) OR (dementia[Title/Abstract]) OR (depression[Title/Abstract]) OR (anxiety[Title/Abstract]) OR (stomach[Title/Abstract]) OR (neur*[Title/Abstract]) OR (mood[Title/Abstract]) OR (supplement[Title/Abstract]) OR (extract[Title/Abstract]) OR (capsules[Title/Abstract]) OR (tablet[Title/Abstract]) OR (powder[Title/Abstract]) OR (memory[Title/Abstract]) OR (mental[Title/Abstract]) OR (neurocognitive[Title/Abstract]) OR (health[Title/Abstract]) OR (cancer[Title/Abstract]) OR (antifungal[Title/Abstract]) OR (antibacterial[Title/Abstract]) OR (anticancer[Title/Abstract]) OR (depressant[Title/Abstract]) OR (antimicrobial[Title/Abstract])).

1. **FIGURES AND TABLES**

**2.1 Figure (1) depicts the PRISMA flowchart for identifying relevant studies.**


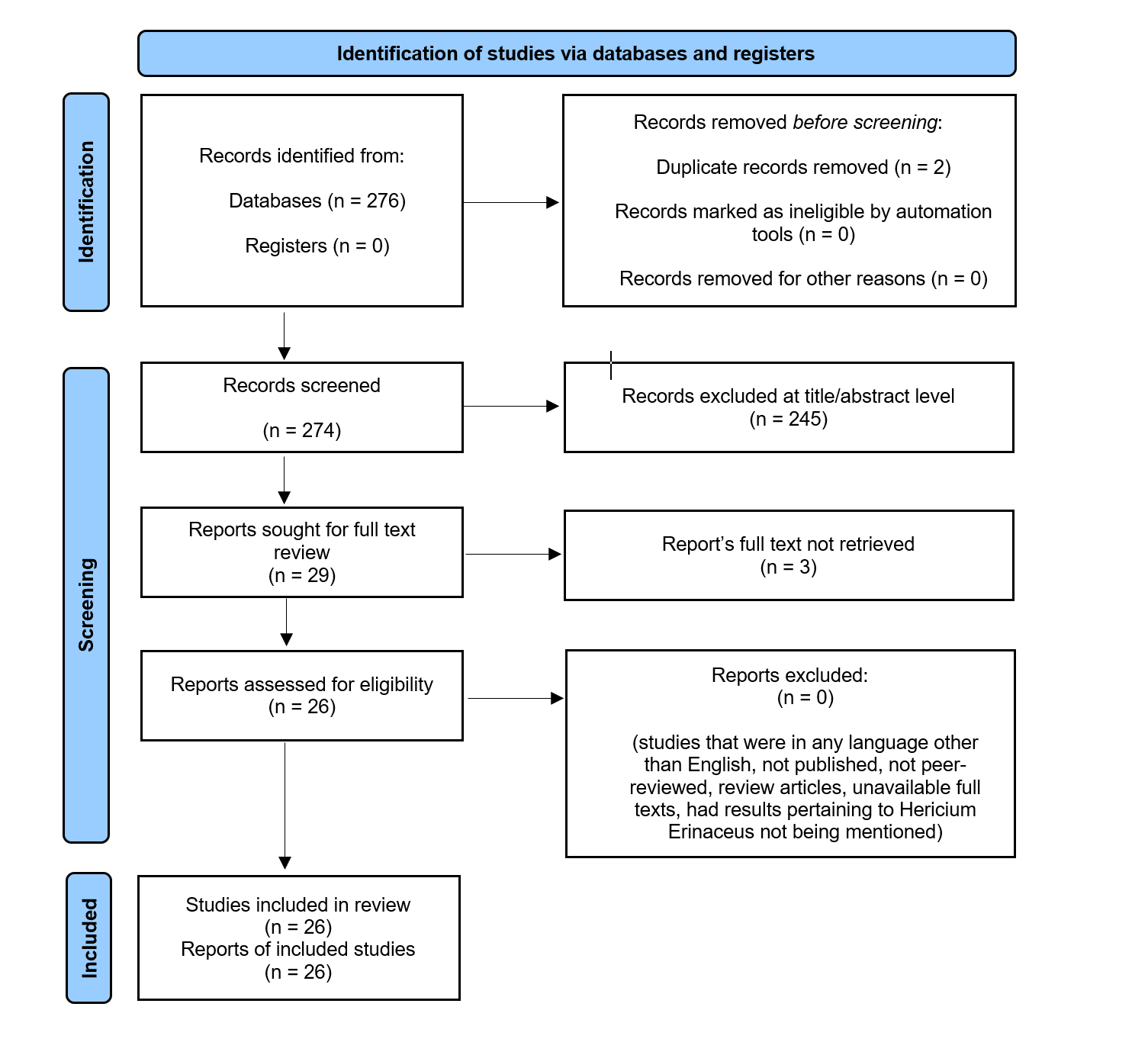


**2.2 Table (1) Patient and Study Characteristics (including all human studies)**

| **Study** | **Design** | **Duration** | **Sample** | **Age (years)** | **Gender** | **Status** | **Study Focus** | **Treatment** | **Side effects** | **Scales** | **References** |
| --- | --- | --- | --- | --- | --- | --- | --- | --- | --- | --- | --- |
| Saitsu et al. (2019), Japan | RCT | 84 days | N = 31  I = 16  C = 15 | I = 61.8±1.7  C = 60.8±2.2 | M = 11  F = 20 | Healthy | Improving cognitive function | HE supplements contained 0.8 g of fruiting powder from HE | None reported | MMSE, Benton visual retention test, S-PA | (15) |
| Nagano et al. (2010), Japan | RCT | 28 days | N = 26  I = 12  C = 14 | I = 41.3±5.6  C = 38.4±4.9 | M = 0  F = 26 | Healthy | Reduction of depression and anxiety | 4 HE cookies daily containing 0.5g of the powdered fruiting body of HE each | Epi-menorrhea | KMI, CES-D. PSQI. ICI | (5) |
| Mori et al. (2009), Japan | RCT | 154 days | N = 30  I = 15  C = 15 | N = 50 to 80 | M = 15  F = 15 | Patient | Improving Mild Cognitive impairment | 4 tablets containing 250mg three times a day | Stomach discomfort and diarrhea | HDS-R | (13) |
| La Monica et al. (2023), USA | RCT | 7-14 days | N = 40  I = 40  C = 40 | N= 34.0±9.5 | M =18  F = 22 | Healthy | Improving cognitive performance | A single dose of 1 g Nordic-grown Lion’s Mane | Headache | Go/No-go, Serial Sevens, N-Back, VAS, SHS | (14) |
| Vigna et al. (2019), Italy | RCT | 120 days | N = 77  I = 40  C = 37 | N = 53.2±0.7 | M = 15  F = 62 | Patient | Improving mood and sleep disorders | 3 HE capsules a day for 8 weeks | None  reported | Zung's Depression and Anxiety scale, Self-Assessment Scale, SCL-90, BES | (28) |
| Xie et al. (2021), China | PCT | 7 days | N = 13  I = 13 | N = 30.0±4.9 | M = 6  F = 7 | Healthy | Influence on serum biochemical markers and gut microbiota | 1g of HE powders 3 times a day | None reported | Chao1, ACE, Simpson, Shannon indices | (17) |
| Li et al. (2020) Taiwan | PCT | 365 days | N = 41  I = 20  C = 21 | I = 74.3±7.15  C = 77.05±8.2 | M = 17  F = 24 | Patients | Prevention of early Alzheimer's Disease | 3 capsules containing 5 mg/g erinacine, an active ingredient, per day | Abdominal discomfort, nausea and skin rash | NPI, CASI, MMSE, IADL | (15) |
| Docherty et al. (2023) United Kingdom | PCT | 28 days | N = 43  I = 22  C = 21 | N = 26.35±6.12 | M = 20  F = 23 | Healthy | Improving cognitive function | Three capsules daily containing HE mushroom = 600mg daily | None reported | COMPASS, S-VAS, VAMS, PSS | (16) |
| Grozier et al. (2022)  United States of America | Cohort study | 28 days | N = 24  I = 12  C = 12 | I = 22.3±3.2  C = 21.8±2.7 | M = 10  F = 14 | Healthy | Impact on metabolic flexibility and cognition | 10 g of HE in 2 muffins per day | None reported | Dual-task challenges consisting of a Stroop Word Challenge with a Mental Arithmetic Challenge and GXT | (18) |
| Nakatsugawa et al. (2003), Japan | Case report | 120 days | N = 1  I = 1 | N = 63 | M = 1  F = 0 | Patient | Not mentioned | HE dry powder extract as diet food | ARDS | N/A | (19) |

**2.3 Table (2) Study characteristics: (Laboratory studies and Computer Analysis)**

| **Study** | **Design** | **Cell type** | **Study Focus** | **Treatment** | **References** |
| --- | --- | --- | --- | --- | --- |
| Tian et al. (2022), China | Laboratory Study | Human Fecal Microbiota | Positive effect on Fecal Microbiota | Three polysaccharides were fractionally precipitated from hot water-soluble HE extracts using 30%, 50%, and 70% ethanol. | (36) |
| Kim et al. (2011) | Laboratory Study | U937 human monocytic leukemia cells | Induced apoptosis of monocytic leukemia | Hot water microwaved 50% ethanol, acidic, and alkaline extracts of the fruitbody of HE at 500 mg ml | (21) |
| Tada et al. (2022), Japan | Laboratory Study | The human monocytic leukemia THP-1 cells | Anti-inflammatory activities by neutralizing lipopolysaccharide-induced pro-inflammatory cytokine production in human monocytes | Ethanol and hot water extracts from HE | (20) |
| Li et al. (2015), Korea | Laboratory Study | HL-60 human acute promyelocytic leukemia and HEL-299 lung fibroblast cells | Anticancer activities against human acute promyelocytic leukemia | Hericerin A and Hericerin J and five known compounds at IC50 concentrations were administered into HL-60 cells | (8) |
| Li et al. (2014), United States of America | Laboratory Study | HepG2 and Huh-7 liver, HT-29 colon, and NCI-87 gastric cancer | Anticancer potential against human gastrointestinal cancers | HTJ5 and HTJ5A in a 100 μl | (7) |
| Zan et al. (2015), China | Laboratory Study | SGC-7901 human gastric carcinoma cells | Inhibits gastric carcinoma via cell cycle arrest and apoptosis | Cell exposure to HEG5 for 1-2 days for absorption | (22) |
| Ellan et al. (2019), Malaysia | Laboratory Study | Human peripheral blood mononuclear | Anti-inflammatory effect on dengue-infected human monocytes | Hot aqueous extraction (HAE) method and aqueous soluble extract (ASE) | (40) |
| Lai et al. (2013), Malaysia | Laboratory Study | neuroblastoma-glioma NG108-15 and lung fibroblast MRC-5 | Neurotrophic properties | HE aqueous extract: 0-1000 μg/mL tested NGF: 5-100 ng/mL tested  Oxidative stress inducer (H2O2/hydrogen peroxide): 100 μM | (41) |
| Zhuang et al. (2023), China | Laboratory Study | human fecal microbiota | Antioxidant and prebiotic activities | HEP A and HEP W polysaccharide dried and ground | (34) |
| Tamrakar et al. (2023), Japan | Laboratory Study | human astrocytoma (BNDF mRNA expression) | Neuroprotective properties | Lipase enzyme treatment of Hericenone C and Deacyl hericenone | (42) |
| Hou et al. (2020), China | Laboratory Study | colorectal cancer: HCT-116 and DLD1 | Induction of apoptosis in human colorectal cancer via ROS regeneration | HE fruiting body extracted and dried | (43) |
| Mori et al. (2009), Japan | Laboratory Study | human astrocytoma | Nerve growth factor-inducing activity in human astrocytoma cells | HE ethanol extract | (13) |
| Kuo et al. (2017) | Laboratory Study | gastric cancer | Inhibition of gastric cancer cell viability and invasiveness | HE ethanol extract isolating erinacine A | (33) |
| Chen et al. (2015), China | Laboratory Study | MCF-7 breast cancer and HeLa | Antioxidant and antiproliferation activities | Water extraction of HE polypeptide | (9) |
| Wang et al. (2019), Taiwan | Laboratory Study | BV2 microglial | Anti-inflammatory effect on microglial cells treated with LPS | Erinacine c through HE ethanol extraction | (44) |
| Sutthibutpong et al. (2024), Thailand | Computer Analysis | N/A | Identifying potential acetylcholinesterase inhibitors | Molecular docking to assess AChE inhibiting potential | (23) |

**2.4 Table (3) Dosage and Toxicology Characteristics**

| **Study** | **Dosage Form** | **Dosage Concentration** | **Dosage Duration** | **Adverse Side Effects** | **Toxicology** | **Compliance** | **Tools Used (Assays)** | **References** |
| --- | --- | --- | --- | --- | --- | --- | --- | --- |
| Saitsu et al. (2019) | Supplement contained fruiting powder | 4/day (3.2 g daily) | 12 weeks | None reported | None reported | High Compliance | As mentioned in (Table 1) | (1) |
| Nagano et al. (2010) | Cookies containing powdered fruiting body | 4/day (2.0 g HE powder/day) | 4 weeks | One participant reported epimenorrhea but was excluded from the sample, not attributed to HE | None reported | High Compliance |  | (5) |
| Mori et al. (2009) | Tablets contained 96% Yamabushitake dry powder | 4 tablets 3/day ( 3.0g / day) | Intake: 16 Follow-Up: 4 | Mild stomach discomfort & diarrhea in 7 Yamabushitake vs 6 placebo; no treatment needed | None reported | High Compliance |  | (17) |
| La Monica et al. (2022) | Capsule containing dried powder of fruiting body | 250mg 4/day ( 1.0 g/day) | 16 weeks | Mild stomach discomfort | None reported | None reported |  | (18) |
| Vigna et al. (2019) | Capsule containing mycelium and fruiting body extract | 400 mg HE mycelium and 100 mg HE fruiting body extract per capsule 3/day (1.5 g/day) | 8 weeks | None reported | None reported | High Compliance |  | (52) |
| Xie et al. (2021) | Dry Powder | 1g 3/day (3.0g/day) | 1 weeks | None reported | None reported | None reported |  | (21) |
| Li et al. (2020) | Dry powder containing fruiting bodies | 350 mg/capsules containing 5 mg/g erinacine A 3/day | 49 weeks | Nausea, abdominal discomfort and nausea | None reported | Average compliance 14.3% dropout |  | (19) |
| Docherty et al. (2023) | Capsule | 600 mg 3 /day 1.8 g/day | 4 weeks | None reported | None reported | High Compliance |  | (20) |
| Grozier et al. (2022) | Muffins containing HE fruiting body powder | 2 muffins/day (10g/day) | 4 weeks | None reported | None reported | High Compliance |  | (22) |
| Nakatsugawa et al. (2003) | Dry powder extract of HE | Not specified (commercial quantities) | 16 weeks | Acute Respiratory Distress Syndrome (ARDS) | Elevated serum SP-A and SP-D indicate lung injury; strong lymphocyte proliferation | Daily use |  | (23) |
| Tada et al. (2022) | Ethanol and hot water extracts from HE | N/A | 24 h | N/A | No direct cytotoxicity to human monocytic cells | N/A | Enzyme-linked immunosorbent assay (ELISA) | (24) |
| Li et al. (2015) | Hericerin A at IC50 concentrations were administered into HL-60 cells | 0.01-100 μM | 72 h | N/A | Hericerin A (IC₅₀ = 3.06 μM) ) showed potent, selective cytotoxicity against HL-60 leukemia cells with minimal toxicity to normal HEL-299 cells (>50 μM) | N/A | MTT cell viability; Flow cytometry (PI, sub‑G1 population); Hoechst 33342 nuclear morphology, Western blot | (8) |
| Li et al. (2014) | HTJ5 and HTJ5A in a 100 μl | 0.156 – 20.0 mg/mL | 72 h | N/A | HTJ5 and HTJ5A exhibited concentration-dependent cytotoxicity in vitro against liver cancer HepG2 and Huh-7, colon cancer HT-29, and gastric cancer NCI-87 cells | N/A | MTT assay | (7) |
| Zan et al. (2015) | Hericium erinaceus polysaccharide-protein HEG-5 | 10-200 μg/mL | 24-48 h | N/A | Dose-dependent reduction in cell viability (up to 93.4% inhibition at 200 µg/mL; induced apoptosis and cell cycle arrest | N/A | MTT assay, Annexin V-FITC and PI Double Staining, Cell Cycle Distribution Assay, Comet Assay, qRT-PCR and Western blot analysis | (26) |
| Ellan et al. (2019) | Hot aqueous extract and aqueous-soluble fraction separated from ethanol extract | 313-1500 µg/mL | 48 h | N/A | Non-cytotoxic up to 1500 µg/mL; no significant inhibition of cytokines at non-cytotoxic doses | N/A | MTT Assay, ELISA Cytokine Assay, and IC₅₀ analysis | (56) |
| Lai et al. (2013) | Aqueous extract (hot water-decocted and freeze-dried) | 1-1000 μg/mL | 24-48 h | N/A | No significant cytotoxicity at the tested doses; protective effects noted under oxidative stress | N/A | MTT Assay, Trypan Blue Assay, TUNEL Assay | (57) |
| Zhuang et al. (2022) | HEP-A and HEP-W | 1-3000 µg/mL | N/A | N/A | No cytotoxic effects reported at tested concentrations | N/A | DPPH, ABTS, radical scavenging assay, Hydroxyl radical-scavenging assay, Simulated saliva gastrointestinal digestion, Human fecal fermentation model, Ion chromatography, HPGFC | (42) |
| Tamrakar et al. (2023) | Ethanol extract of hericenone C and its deacylated derivative (deacyl hericenone) | 1.6-12.5 µg/mL | 24 h | N/A | No indications of cytotoxicity at tested doses; deacyl hericenone showed enhanced protective effects over parent compound in oxidative stress model | N/A | LC–QTOF–MS, ¹H‑NMR, BDNF mRNA expression assays, Oxidative stress cell viability assay | (58) |
| Hou et al. (2020) | HE fruiting body polysaccharides | Not specified | 12-120 h | N/A | Selective cytotoxicity to cancer cells via reactive oxygen species-mediated caspase-9 pathway and intrinsic apoptotic pathway | N/A | Cell Viability Assay, Reactive Oxygen Species Measurement Assay, Mitochondrial Membrane Potential Assay, Western Blot | (59) |
| Mori et al. (2008) | Ethanol extract of HE fruiting bodies | 50-250 µg/mL | 3-168 h | N/A | No cytotoxicity evident at tested doses | N/A | MTT Assay, RT-PCR, Enzyme Immunoassay of NGF | (50) |
| Kuo et al. (2017) | Erinacine A | 1-10 µM | 24 h | N/A | Dose-dependent cytotoxicity and apoptosis | N/A | MTT Assay, Boyden Chamber Assay, Annexin V–FITC/ Propidium Iodide staining, Proteomics Analysis, Western Blot | (36) |
| Chen et al. (2015) | Water-extracted and alcohol-precipitated polysaccharides from HE | 50-200 µg/mL | 24 h | N/A | Dose-dependent inhibition of cancer cell proliferation | N/A | MTT Assay, DPPH· Radical-Scavenging Assay, Hydroxyl Radical-Scavenging Assay | (9) |
| Wang et al. (2019) | Erinacine C | 0.1–10 μM | 25 h | N/A | No cytotoxicity observed at 0.1–2.5 µM | N/A | Cell Growth Analysis (Hemocytometer), Nitric Oxide Assay, ELISA, Western Blot | (60) |
| Tian et al. (2022) | Three polysaccharides from hot water-soluble extracts of HE | 8 mg/mL | 24 h | N/A | No direct report of toxicity or adverse effects | N/A | No toxicology assays performed | (43) |
| Kim et al. (2011) | Hot water, ethanol, acidic, and alkaline extracts of the fruiting body of HE | 500 μg/mL | 2-48 h | N/A | HWE and MWE decreased viability of U937 cells; ACE and AKE (controls) were not cytotoxic | N/A | MTT assay to assess cell viability, Caspase activity assays (caspase-3, -8, -9), Mitochondrial membrane potential (MMP) assay, | (25) |

- 1. **Risk of Bias Assessment**

**2.4.1 Table (4) Cochrane risk of bias (RoB 2) tool for clinical trials**

| **Cochrane Risk of Bias (RoB 2) Tool** | | | | | | |
| --- | --- | --- | --- | --- | --- | --- |
| **Study** | **Bias from Randomization** | **Bias from Deviations from Intended Interventions** | **Bias from Missing Data** | **Bias in Measurement of Outcomes** | **Bias in Selection of the Reported Result** | **Overall Risk of Bias** |
| Saitsu et al. (2019) | Low | Low | Low | Low | Low | Low |
| Nagano et al. (2010) | Some concerns | Low | Low | Low | Low | Some concerns |
| Mori et al. (2009) | Some concerns | Low | Low | Low | Low | Some concerns |
| La Monica et al. (2022) | Low | Low | Low | Low | Low | Low |
| Vigna et al. (2019) | Low | Some concerns | Low | Low | Low | Some concerns |
| Xie et al. (2021) | High | Some concerns | Low | Low | Low | High |
| Li et al. (2020) | Low | Low | Low | Low | Low | Low |

**2.4.2 Table (5) Adapted from SYRCLE’s RoB tool for laboratory studies**

| **Adapted from SYRCLE’s RoB tool** | | | | | |
| --- | --- | --- | --- | --- | --- |
| **Study** | **Selection Bias** | **Performance Bias** | **Attrition Bias** | **Reporting Bias** | **Overall Risk of Bias** |
| Tada et al. (2022) | Low | Some concerns | Low | Low | Some concerns |
| Li et al. (2015) | Low | Some concerns | Low | Low | Some concerns |
| Li et al. (2014) | Low | Some concerns | Low | Low | Some concerns |
| Zan et al. (2015) | Low | Some concerns | Low | Low | Some concerns |
| Ellan et al. (2019) | Low | Some concerns | Low | Low | Some concerns |
| Lai et al. (2013) | Low | Some concerns | Low | Low | Some concerns |
| Zhuang et al. (2022) | Low | Some concerns | Low | Low | Some concerns |
| Tamrakar et al. (2023) | Low | Low | Low | Low | Low |
| Hou et al. (2020) | Low | Some concerns | Low | Low | Some concerns |
| Mori et al. (2008) | Low | Some concerns | Low | Low | Some concerns |
| Kuo et al. (2017) | Low | Low | Low | Low | Low |
| Chen et al. (2015) | Low | Some concerns | Low | Low | Some concerns |
| Wang et al. (2019) | Low | Some concerns | Low | Low | Some concerns |
| Tian et al. (2022) | Low | Some concerns | Low | Low | Some concerns |
| Phil Kim et al. (2011) | Low | Low | Low | Low | Low |

**2.4.3 Table (6) ROBINS-I tool for observational study**

| **ROBINS-I Tool** | | | | | | | | |
| --- | --- | --- | --- | --- | --- | --- | --- | --- |
| **Study** | **Bias due to Confounding** | **Bias in Classification of Interventions** | **Bias due to Selection** | **Bias due to Deviations from Intended Interventions** | **Bias due to Missing Data** | **Bias in Measurement of Outcomes** | **Bias in Selection of the Reported Result** | **Overall Risk of Bias** |
| Grozier et al. (2022) | Moderate | Low | Low | Moderate | Low | Low | Low | Moderate |

**2.4.4 Table (7) Institute of Health Economics (IHE) Case Report Appraisal Tool**

| **Institute of Health Economics (IHE) Case Report Appraisal Tool** | | | | | | | | | | | | |
| --- | --- | --- | --- | --- | --- | --- | --- | --- | --- | --- | --- | --- |
| **Study** | **Objective clearly stated** | **Prospective conduct** | **Patient population described** | **Eligibility criteria clear** | **Intervention details clear** | **Co-interventions described** | **Outcome measures relevant and clear** | **Outcome assessors blinded** | **Follow-up adequate** | **Adverse events reported** | **Conclusions supported by data** | **Competing interests declared** |
| Nakatsugawa et al. (2003) | Yes | Yes | Yes | Yes | Yes | Yes | Yes | Unclear | Yes | Yes | Yes | Unclear |

**2.4.5 Table (8) Risk of bias assessment for computer analysis**

| **Study** | **Transparent Model Description** | **Data Source Reliability** | **Model Validation** | **Sensitivity Analysis** | **Reproducibility (Code/Data Sharing)** | **Selective Reporting** | **Overall Risk of Bias** |
| --- | --- | --- | --- | --- | --- | --- | --- |
| Sutthibutpong et al. (2024) | Low risk | Low risk | Low risk | Some concerns | Some concerns | Low risk | Some concerns |
